# Tumor Mutational Burden as a Potential Biomarker for Immunotherapy in Pancreatic Cancer: Systematic Review and Still-Open Questions

**DOI:** 10.3390/cancers13133119

**Published:** 2021-06-22

**Authors:** Rita T. Lawlor, Paola Mattiolo, Andrea Mafficini, Seung-Mo Hong, Maria L. Piredda, Sergio V. Taormina, Giuseppe Malleo, Giovanni Marchegiani, Antonio Pea, Roberto Salvia, Valentyna Kryklyva, Jae Il Shin, Lodewijk A. Brosens, Michele Milella, Aldo Scarpa, Claudio Luchini

**Affiliations:** 1 ARC-Net Research Center, University and Hospital Trust of Verona, 37134 Verona, Italy; ritateresa.lawlor@univr.it (R.T.L.); andrea.mafficini@univr.it (A.M.); marialiliana.piredda@univr.it (M.L.P.); sergiovincenzo.taormina@univr.it (S.V.T.); 2 Department of Diagnostics and Public Health, Section of Pathology, University and Hospital Trust of Verona, 37134 Verona, Italy; paola.mattiolo@studenti.univr.it; 3 Asan Medical Center, Department of Pathology, University of Ulsan College of Medicine, Seoul 05505, Korea; shong28@amc.seoul.kr; 4 Department of Surgery, The Pancreas Institute, University and Hospital Trust of Verona, 37134 Verona, Italy; giuseppe.malleo@univr.it (G.M.); giovanni.marchegiani@univr.it (G.M.); antonio.pea@univr.it (A.P.); roberto.salvia@univr.it (R.S.); 5 Department of Pathology, Radboud Institute for Molecular Life Sciences, Radboud University Medical Center, 6525 GA Nijmegen, The Netherlands; valentyna.kryklyva@radboudumc.nl (V.K.); l.a.a.brosens@umcutrecht.nl (L.A.B.); 6 Department of Pediatrics, Yonsei University College of Medicine, Seoul 120-752, Korea; SHINJI@yuhs.ac; 7 Department of Pathology, University Medical Center Utrecht, Utrecht University, 3584 CX Utrecht, The Netherlands; 8 Department of Medicine, Section of Oncology, University and Hospital Trust of Verona, 37134 Verona, Italy; michele.milella@univr.it

**Keywords:** tumor mutation burden, TMB, TML, PD-1, PD-L1, pancreatic cancer, immunotherapy

## Abstract

**Simple Summary:**

Tumor mutational burden (TMB) represents the number of mutations per megabase (muts/Mb) harbored by tumor cells in a given neoplasm, and can be determined with next-generation sequencing. High values are an indicator of potential response to immunotherapy. With this systematic review, we assessed its role in pancreatic ductal adenocarcinoma (PDAC). Our main findings can be summarized as: (i) high-TMB can be found in about 1% of PDAC; (ii) it is associated with mucinous/colloid and medullary histology; (iii) high-TMB PDAC frequently harbor other actionable alterations, with microsatellite instability as the most common; (iv) immunotherapy has shown promising results in high-TMB PDAC.

**Abstract:**

Tumor mutational burden (TMB) is a numeric index that expresses the number of mutations per megabase (muts/Mb) harbored by tumor cells in a neoplasm. TMB can be determined using different approaches based on next-generation sequencing. In the case of high values, it indicates a potential response to immunotherapy. In this systematic review, we assessed the potential predictive role of high-TMB in pancreatic ductal adenocarcinoma (PDAC), as well as the histo-molecular features of high-TMB PDAC. High-TMB appeared as a rare but not-negligible molecular feature in PDAC, being present in about 1.1% of cases. This genetic condition was closely associated with mucinous/colloid and medullary histology (*p* < 0.01). PDAC with high-TMB frequently harbored other actionable alterations, with microsatellite instability/defective mismatch repair as the most common. Immunotherapy has shown promising results in high-TMB PDAC, but the sample size of high-TMB PDAC treated so far is quite small. This study highlights interesting peculiarities of PDAC harboring high-TMB and may represent a reliable starting point for the assessment of TMB in the clinical management of patients affected by pancreatic cancer.

## 1. Introduction

Pancreatic ductal adenocarcinoma (PDAC) is a highly malignant disease, with 5-year overall survival <5% [1]. One of the most promising discoveries in the era of precision oncology is represented by immunotherapy [2]. The so-called “check-point inhibitors” can also be administered to patients with PDAC, and especially to those harboring microsatellite instability (MSI). Next-generation sequencing (NGS) is gaining a prominent role for selecting treatment options. One of the biomarkers that can be investigated with NGS is tumor mutational burden (TMB) [3,4].

Tumor mutational burden (TMB, also called tumor mutational load) is an emerging biomarker in cancer therapy. It represents an index indicating the number of mutations per megabase (muts/Mb) harbored by tumor cells in a given neoplasm [3]. TMB is considered high if it exceeds a predetermined threshold, which has been set around 17–20 muts/Mb [4]. However, recent studies have pointed out that this cut-off may widely vary based on differing factors, including tumor type. Marabelle et al. used 10 muts/Mb as cut-off when analyzing different solid tumors [5], while Schrock et al. identified in 37 muts/MB the optimal cut-off in the specific case of colorectal cancers [6]. Of note, Samstein et al. suggested that the ideal TMB-high group should overlap with the highest mutational load quintile in each histology [7]. In cases of high values, TMB is a predictive biomarker, potentially indicating a high rate of response to immunotherapy [8]. 

The biological explanation behind this assumption is that tumor cells with a high-TMB tend to produce more immunogenic neoantigens, whose recognition by host T cells, above all T cytotoxic lymphocytes, is one of the most important aspects in predicting immunotherapy response. Of all available immunotherapies, a high-TMB is strictly associated with response to anti-PD-1 therapies, as clarified by a pooled analysis of 27 tumor types [9,10]. Interestingly, TMB is not the only important indicator of immunotherapy response; indeed, the expression of Programmed Death-Ligand 1 (PD-L1) by tumor cells and the presence of microsatellite instability (MSI) are also predictive biomarkers. The fact that a high-TMB can exist also in the absence of these other biomarkers, as already demonstrated, indicates that the determination of TMB could increase the population that may benefit from immunotherapy [9,10,11,12,13,14,15,16,17].

TMB can be determined by different NGS methods, but the optimal approach calculates TMB based on exome-wide sequencing analysis (WES) covering approximately 30 Mb. However, this method is challenging to adopt in daily clinical practice, due to high costs and long turnaround times. As a consequence, an increasing number of studies has tried to demonstrate whether targeted NGS gene panels might determine TMB with reliable precision [15,18,19,20]. While initial studies have shown that targeted NGS gene panels offer reliable estimates of TMB, substantial concerns regarding the stochastic error associated to limited gene panel sequencing have been raised [15,21,22,23,24,25,26]. It is also still under debate whether recurrent or driver mutations should be excluded from the TMB determination [12,18]. The precision of targeted sequencing (limited gene panel) based calculation of tumor mutational burden (tsTMB) may be also highly affected by pre-analytical factors, such as: (i) the amount of genome interrogated; (ii) the read depth; (iii) the presence of intratumor heterogeneity, with potential over and underestimation of the real TMB; (iv) the tissue-fixation methodologies when using formalin-fixed paraffin embedded tissue (FFPE), and (v) sample age, in particular for FFPE samples [8,26,27]. Some of these factors can impact TMB reproducibility, even when using the same assay [8]. Another significant issue is represented by the lack of standardization of the genes of interest, which can vary greatly among commercial and custom panels [3,11,12,18,19].

Extensive investigations on tumors of different origins highlighted how high-TMB may be observed in almost all cancer types [3]. Recent evidence has clarified that several factors may determine a high-TMB in different neoplasms, including polymerase-epsilon (*POLE*) mutations [28], environment-based etiologies such as tobacco smoke, polycyclic aromatic hydrocarbons and UV exposure [19,27,29,30], or the presence of MSI [4,6]. Currently, TMB-based approved immunotherapy approaches include non-small cell lung cancers [31], bladder cancers [32], and malignant melanomas [33] with TMB cut-off of 10 mutations per Mb [5,30]. 

Interestingly, little is known regarding the prevalence and the potential prognostic/predictive roles of TMB in pancreatic cancers, although preliminary data suggests a possible role of immunotherapy in selected cases of PDAC [4,5]. The aim of the current study is to provide a systematic review of the current knowledge of TMB in the context of pancreatic cancer, highlighting new possible horizons for immunotherapy in the context of this highly malignant neoplasm.

## 2. Results

### 2.1. Search Results

Altogether, the search yielded 123 non-duplicated articles. After excluding 102 articles based on title/abstract review, 21 articles were retrieved for full text review of which 13 studies were included in this systematic review (Figure S1) [34,35,36,37,38,39,40,41,42,43,44,45,46].

### 2.2. Clinico-Pathologic Characteristics

The 13 articles selected for this systematic review reported a total number of 47 PDAC with high-TMB from 1998 PDAC that were tested for TMB (Table 1); extracted specific data from studies dealing with TMB prevalence indicates that 1.1% of all PDAC harbored a high-TMB (using the definitions adopted by papers’ authors). The majority of cases affected the pancreatic head (64%), and the most common histology was conventional ductal adenocarcinoma (80%) (Table 1).

IPMN-associated mucinous-colloid PDAC represented 14% of all cases, while particular variants such as medullary PDAC and signet ring PDAC were not so uncommon (4% and 2%, respectively). Comparing this prevalence with well-established and large series/datasets of PDAC [47,48], high-TMB cases demonstrated a higher prevalence of mucinous-colloid and medullary carcinomas, reaching statistically significant differences (*p* < 0.01, Fisher’s exact test). Regarding TNM staging, the majority of cases (60%) were stage II, followed by stage IV (33%) and stage III (7%) (Table 1). 

### 2.3. Molecular Data of High-TMB PDAC

**Figure 1:**
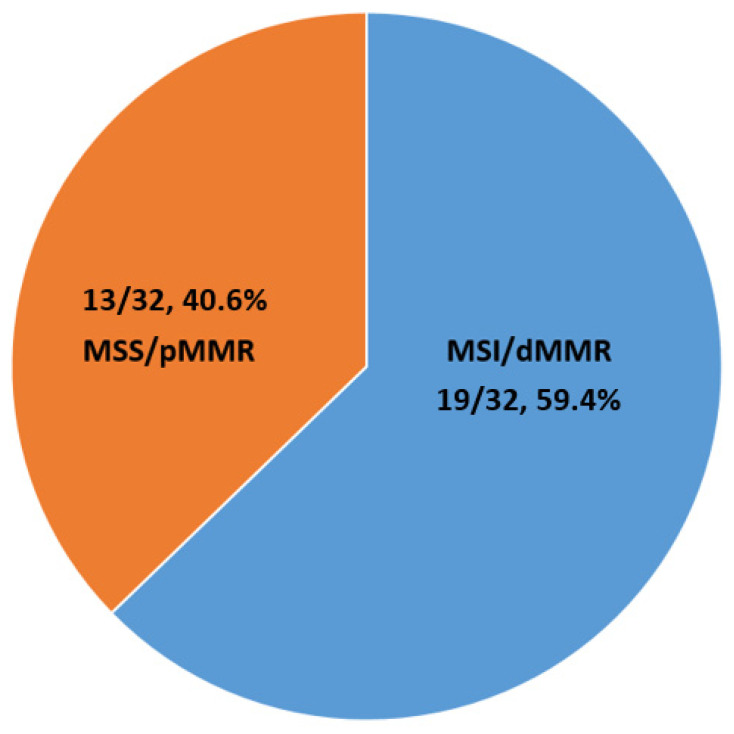
Summarizing figure highlighting the prevalence of MSI/dMMR in high-TMB PDAC. Abbreviations: MSI: microsatellite instability; dMMR: defective mismatch repair; MSS: microsatellite stable; pMMR: proficient mismatch repair.

The mean value of TMB of the investigated high-TMB cases was 37.6 mut/Mb. The highest value was observed in the single *POLE*-mutated PDAC (111 mut/Mb, which is considered a hyper-ultra-mutated TMB) [39]. The majority of high-TMB cases (59.4%) also harbored MSI/dMMR (Figure 1).

One of these also displayed an additional actionable *BRAF* V600E alteration [45]. In studies for which complete molecular profile data was available, microsatellite-stable (MSS) cases frequently showed the presence of potentially actionable targets. Indeed, two cases harbored an *ERBB2* amplification/mutation [34,39], five cases showed *BRCA2* mutations [37,43] and one case displayed a *POLE* mutation [39] (Table 2).

### 2.4. Response to Immunotherapy of High-TMB PDAC

Data on therapeutic approaches and survival is very inconsistent, reflecting non-standardized therapeutic regimens for cases of high-TMB PDAC, in addition to the retrospective nature of several of the studies examined. Regarding immunotherapy, a total of eight PDAC with high-TMB received anti-PD1 therapy (Table 1). Of these, five cases showed a wide spectrum of partial response, one case showed stable disease after 30 months of therapy, and two cases showed complete response. These two cases were both also MSI/dMMR [35,41]; one patient was alive without disease more than 5 years after surgical resection, and the other patient showed a complete response more than 2 years after surgery.

## 3. Discussion

**Figure 2:**
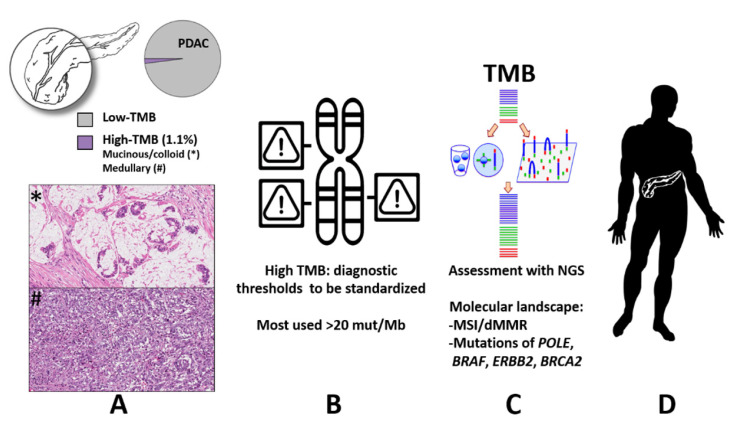
Summarizing figure highlighting the complex landscape related to tumor mutational burden and its potential roles as a biomarker in precision oncology. (**A**) High-tumor mutational burden is relatively rare in pancreatic ductal adenocarcinoma (1.1%), and is enriched in mucinous/colloid and medullary histology (original magnification 10×); (**B**) the most used threshold to distinguish high- vs. low-tumor mutational burden is 20 mutations/Mb; (**C**) tumor mutational burden is assessed with next-generation sequencing, and pancreatic cancers with high-tumor mutational burden display a peculiar molecular landscape, including microsatellite instability/defective mismatch repair, and mutations affecting *POLE*, *BRAF*, *ERBB2* and *BRCA2* genes; (**D**) the routine assessment of tumor mutational burden should be intended as an important step towards the implementation of precision oncology for patients affected by pancreatic cancer. Abbreviations: TMB: tumor mutational burden; mut/Mb: mutations per megabase; NGS: next-generation sequencing; MSI: microsatellite instability; dMMR: defective mismatch repair.

In this systematic review, we reported clinic-pathologic, molecular, and therapeutic data derived from a total of 47 PDAC with high-TMB. Although 47 cases of PDAC represent a limited sample size, the prevalence of high-TMB represents about 1.1% of all PDAC tested for this variable, which while low is a non-negligible percentage of cases. The most important findings and implications are summarized in Figure 2.

For PDAC in general, high-TMB PDAC mainly involve the pancreatic head, with more than 60% of cases resulting in this location. In terms of histologic subtypes, there is a higher prevalence of mucinous-colloid and medullary histology, which usually represent less than 2% of all PDAC [47,48,49,50] but, in the case of high-TMB, PDAC represent 14% and 4% of all cases, respectively. These differences are statistically significant and, at least in part, reflect the association of high-TMB with MSI/dMMR in PDAC, where these histological variants have already been demonstrated to be more prevalent [35,51,52,53]. However, as a significant proportion (about 40% of cases) of high-TMB PDAC do not harbor MSI/dMMR, the histological differences observed for this molecular subgroup of PDAC can be considered a peculiarity also of this genetic condition. 

Along this line, the case described by Kryklyva et al. of a PDAC with the highest reported TMB (111 muts/Mb) [39] is noteworthy: the case was a medullary PDAC and the high-TMB was due to a *POLE* mutation and not to MSI/dMMR. Furthermore, as observed for other tumor types, pathogenic mutations affecting *POLE* also seem to be associated with a very high-TMB, defined as hyper-ultra-mutated phenotype in pancreatic cancer [4,54,55]. It is worth noting that *POLE*-mutated cases have shown a high rate of response to immunotherapy in tumors of other sites, such as colorectal and endometrial cancers [6,56,57], and as such, this should also be explored for PDAC.

In addition to high-TMB, it is of interest reporting that the PDAC evaluated harbor actionable molecular alterations in addition to MSI/dMMR, including *BRAF* mutation, *ERBB2* alterations, and *BRCA2* mutations, which are also found in MSS cases. These findings highlight the presence of still-open questions in the context of extensive tumor molecular profiling. In cases of simultaneous coexistence of high-TMB with another molecular actionable alteration, which is the preferred molecular-based option? Immunotherapy vs. another molecularly-tailored treatment? Further research is needed to answer this fundamental question. 

It should also be noted that the study that identified the coexistence of *BRCA2* mutations with high-TMB used a low threshold (8 muts/Mb) to define high-TMB, and therefore, these cases may be erroneously considered as concomitant [43]. That said, it is also true that alterations affecting the homologous recombination machinery have been already associated with high-TMB in other tumors [58,59]. However, PDAC harboring *BRCA*-genes mutations represent one of the very few PDAC molecular sub-groups with already established effective therapeutic strategies, which includes platinum-based chemotherapy and PARP-inhibitors [60,61,62]. Thus, in such cases, in the presence of high-TMB, the potential role of immunotherapy may be considered at a later stage for non-responders or in the case of disease progression. Another important consideration is the need for more extensive molecular profiling for all PDAC cases with high-TMB, given the potential presence of additional actionable molecular alterations in this tumor category. In this context, it is also of importance considering the different thresholds used by different studies to assess high-TMB. The most used was 20 mut/Mb, but a standard consensus on this point is still lacking, representing another open question in this topic that needs an urgent answer. Notably, in an ongoing phase II clinical trial, the CCTG PA.7 study, the investigators adopted the value of 9 mut/Mb as threshold, yielding 4.6% high-TMB patients. This study investigated whether adding durvalumab to standard chemotherapy might be better in first-line metastatic PDAC patients. Although first evidence did not show a general benefit for such a combination, the potential role of high-TMB in identifying selected patients for this therapeutic approach is currently under investigation [63].

Moreover, recent data suggests that immunotherapy may be less active in MSI/dMMR PDAC compared with other tumor types [5]. Indeed, in an update of KEYNOTE-158, a phase II trial with anti-PD-1 immunotherapy after progression or intolerance to standard regimens in non-colorectal MSI/dMMR cancers, the response rate was significantly lower for MSI/dMMR PDAC than other cancers harboring the same genetic alteration [5]. Notably, among MSI/dMMR colorectal cancer, the coexistence of high-TMB seemed to identify the best responders to immunotherapy [6]. Thus, the assessment of TMB should also be coupled with MSI/dMMR determination in patients with pancreatic cancer, verifying whether this variable may have a role in refining the identification of immunotherapy-responders. To complete the overview on this scenario, it should be noted that some efforts including clinical studies have been already performed, aiming at increasing the opportunities of immunotherapy in PDAC [64,65], but up to date, the most convincing potentialities still belong to MSI/dMMR and high-TMB.

Regarding specific data on immunotherapy responses in PDAC patients with high-TMB, this systematic review clearly showed a very inconsistent scenario, reflecting the lack of standardized therapeutic regimens as well as the retrospective nature of several of the studies reviewed. Notably, analysis of the literature presented eight cases of high-TMB PDAC that received immunotherapy, all having anti-PD1 therapy. Five of these showed a partial response, one case showed stable disease after 30 months of therapy, and two cases, both also MSI/dMMR, showed a complete response. This data indicates that the presence of high-TMB may be of great help in identifying potential immunotherapy responders; at the same time, the small sample size does not permit any definitive conclusions.

This study does have some limitations, which are largely reflected by the limitations of the primary studies. The studies used different methodologies to evaluate TMB, in addition to different thresholds for high-TMB classification, and as such, may have generated some inconsistencies. However, TMB assessment using multi-gene panel NGS approaches, the most frequently used methods in the majority of the studies analyzed, is in line with the methodologies most available and applied in molecular pathology routine activity. Furthermore, some studies did not report data on histology or grading, thus further input are needed to confirm our preliminary findings. At last, while a sample size of 47 high-TMB PDAC does not represent a large cohort of cases, this study can be seen as a robust starting point to guide further investigations on the potential role of immunotherapy for PDAC patients, answering the important open questions above indicated.

## 4. Materials and Methods

This systematic review adhered to the MOOSE guidelines and PRISMA statement [66,67], following a predetermined protocol. 

### 4.1. Inclusion and Exclusion Criteria

Studies were eligible if they met the following criteria: (1) original and complete study on human pancreatic cancer; (2) clear description of the method(s) used for testing TMB; (3) clear report of the total number of cases of pancreatic cancer with high-TMB; (4) publication in a peer review journal in the English language. Exclusion criteria were: (1) cancers from organs other than pancreas; (2) non-invasive cancer (e.g., IPMN); (3) no data regarding TMB evaluation; (4) preliminary abstracts and in vitro or animal studies.

### 4.2. Data Sources and Literature Search Strategy

Two investigators (R.T.L., C.L.) independently searched PubMed, SCOPUS, and Embase up to 01/31/2021. The search terms used in PubMed included combinations of the following keywords: (“tumor mutation burden” OR “tumor mutational burden” OR “tumour mutation burden” OR “tumour mutational burden” OR “tumor mutation load” OR “tumor mutational load” OR “tumour mutation load” OR “tumour mutational load” OR “TML” OR “TMB”) AND (“pancreatic” OR “pancreas”). A similar search was carried out in SCOPUS and Embase. We also considered the reference lists of all included articles and of previous related reviews. 

### 4.3. Study Selection 

Following the searches as outlined above, after removal of duplicates, two independent reviewers (P.M., C.L.) screened titles and abstracts of all potentially eligible articles. The two authors applied the eligibility criteria, reviewed the full texts, and a final list of selected articles was reached through consensus with a third author (A.S.).

### 4.4. Data Extraction, Synthesis, and Statistical Analysis

Two authors were involved in data extraction in a standardized Microsoft Excel database. Specifically, one author (C.L.) extracted data from the included articles and a second independent author (A.S.) validated the data. For each article, information about authors, year of publication, country of origin of the analyzed cohort, number of patients, tumor site in the pancreas, tumor histology, TMB value, method of TMB assessment, MMR/MSI status, data on molecular profile, pathological TNM, therapeutic strategies and survival outcomes was extracted. Data on histology were compared with large series/datasets of PDAC to find potential differences or peculiarities. Finally, all extracted data were reported and summarized in Table 1, and then analyzed, interpreted, and discussed by all authors. 

## 5. Conclusions

In conclusion, this systematic review has highlighted that high-TMB PDAC represents a rare but not-negligible alteration, being present in about 1% of all pancreatic cancers. These tumors display peculiar features, from both a histological and molecular points of view. They are enriched in mucinous/colloid and medullary variants, and in about 60% of cases are associated with MSI/dMMR. Given the remarkable presence of actionable molecular alterations, this PDAC subgroup should be extensively investigated by NGS. 

Preliminary data shows promising results for immunotherapy in this tumor setting. Further studies are needed along this line to explore the most important and still-open questions in this context, such as: (1) What is the correct timing for immunotherapy administration in cases of PDAC with high-TMB? (2) In the case of MSI/dMMR PDAC, would assessment of TMB help to improve the identification of immunotherapy responders? (3) In the case of co-occurrence of high-TMB with another actionable alterations, which is the best option for therapeutic selection? 

Overall, based on this systematic review, it is time to consider TMB as a potential biomarker to improve the management of patients with pancreatic cancer.

## Figures and Tables

**Table 1:** Summarizing table of the main clinic-pathologic features of all PDAC tested for tumor mutational burden.

Author, Year	Country	N of Cases with High TMB	Tumor Site in the Pancreas	Tumor Histology	TNM
Chen, 2019 [34]	China	1	Head	Moderately differentiated PDAC	IV
Grant, 2020 [35]	Canada	9	6 Head, 1 Body-Tail, 2 NA	2 cases: IPMN-associated PDAC, 3 PDAC NOS (2 with medullary areas), 1 anaplastic PDAC, 3 histology NA	1 case IIA, 5 cases IIB, 1 case III, 2 cases IV
Humphris, 2017 [36]	Australia	5	NA	1 case G4, 3 G2, 1 signet-ring	NA
Hu, 2018 [37]	USA	7	1 head/body, 2 body-tail, 1 head, 3 NA	2 conventional, 4 mucinous/colloid IPMN-associated, 1 medullary	1 case pT4, 1 case stage IIB, 2 cases stage IV, remaining cases NA
Nagashima, 2019 [38]	Japan	0/131	NA	NA	NA
Kryklyva, 2020 [39]	The Netherlands	1	Head	Medullary PDAC	IIA
Kimura, 2020 [40]	Japan	0/17	NA	NA	NA
Ngo, 2020 [41]	USA	1	Tail	PDAC	IIB
Overman, 2020 [42]	USA	0/2	NA	Only 2 long survivor PDAC have been tested for TMB: they were TMB-low and MSS	NA
Park, 2020 [43]	USA	5/50 HRD PDAC	NA	PDAC	
Salem, 2018 [44]	USA	12/870	NA	PDAC	NA
Singhi, 2019 [45]	USA	5/1021	NA	PDAC	NA
Tuli, 2019 [46]	USA	1	NS	PDAC	NS
TOTAL	-	22/2091 (1.1%)	H: 64%, BT: 36%	80% PDAC, 14% IPMN-associated mucinous/colloid; 4% medullary; 2% signet ring	I: 0%; II: 60%; III: 7%; IV: 33%

Abbreviations: PDAC: pancreatic ductal adenocarcinoma; N: number; TMB: tumor mutation burden; mut: number of mutations; Mb: megabase; TNM: pathological assessment of “tumor nodal metastasis” staging; NA: data not available; NS: data not specified in the study.

**Table 2:** Summarizing table of the main molecular features and survival data of all PDAC tested for tumor mutational burden.

Author, Year	N of Cases with High TMB ^#^	TMB Value (mut per Mb)	Method of NGS/TMB Measurement	MMR/MSI Status *	Data on Molecular Profile	Summary of Therapeutic Strategies	Data on Survival
Chen, 2019 [34]	1	14.9	NGS on cell-free DNA from blood; 156 genes-panel, Illumina platform	Stable (IHC)	*ERBB2* amplification and mutation. Other mutations in *UGTIA1*, *GSTPI* and *MTHFR*	RCT (gemcitabine); trastuzumab; erlotinib; antiangiogenic therapy; immunotherapy (pembrolizumab)	Dead 20 months after diagnosis
Grant, 2020 [35]	9	Median: 25.93	WGS, Illumina platform	All MSI (paper on MSI PDAC)	More *JAK1* and *ACV2RA*, Less *KRAS* and *SMAD4* mutations	Surgical resection except of IV stage patients. Two patients had adjuvant IT (1 partial response, 1 disease free), 2 no AT (1 alive with disease, recurrence in mesentery, at 33.1 months, 1 alive without disease at 104 months); 3 NAT (2 dead, 1 alive, received adjuvant IT), 3 AT (gemcitabine, alive and disease- free after 42, 44, and 107 months, respectively)	
Humphris, 2017 [36]	5	Threshold 12 mut/Mb, Mean value: 31.8	WGS	4 MSI, 1 microsatellite stable	1 case with somatic homozygous deletion of *MSH2*, 1 case *MHL1* hypermethylation, 1 case *MSH2* desrupting rearrangement, 1 case *MSH2* somatic splice site, 1 case unknown	NA	NA
Hu, 2018 [37]	7	Threshold 12 mut/Mb, Mean value: 51,3 for cases with MSI/dMMR, 54 for a pathogenic *BRCA2* mutated PDAC	MSK-IMPACT (341 cancer-associated gene panel)	6 MSI/dMMR PDAC	2 cases with germline *MSH2* mutations, 2 with germline *PMS2* mutation, 1 germline *MLH1*, 1 germline *MSH6,* 1 unspecified pathogenic *BRCA2* mutation	MSI/dMMR cases: 1 case FOLFIRINOX and FOLFIRI in a neoadjuvant context, followed IT with anti-PD-1: partial response for over 22 months; 1 case: distal pancreatectomy, adjuvant gemcitabine, GVAX, progression and new diagnoses of bladder and gastric cancer, complete response with IT with anti–PD-1 therapy after 2 years; distal pancreatectomy and hemicolectomy (concomitant colorectal cancer); adjuvant 5-fluorouracil and RT: no recurrence in 26 years after surgery; 1 patient pancreaticoduodenectomy, adjuvant gemcitabine, capecitabine, RT, then progression, FOLFOX: no recurrence after 36 months; 1 patient: IT with anti-PD-1 therapy: significant but unspecified regression; 1 case: metastatic PDAC, FOLFIRINOX, gemcitabine, and nab-paclitaxel, then IT with anti-PD-L1; then progression to death 30 months after the diagnosis.	
Nagashima, 2019 [38]	0/131	NA	WES	NA	NA	NA	NA
Kryklyva, 2020 [39]	1	111	NGS with 30 genes-panel, Illumina platform	Stable (IHC and NGS)	*POLE*, *ERBB2*, *GNAS*, *KRAS*, *MAP2K1*, *TP53*	Surgical resection, no AT	Alive free of disease 5 years after surgery
Kimura, 2020 [40]	0/17	NA	WES	NA	NA	NA	NA
Ngo, 2020 [41]	1	High (NS)	Not specified	MSI (Lynch syndrome)	*MSH2* germline mutation	Lynch syndrome; NAT with gemcitabine and nab- paclitaxel, surgical resection, AT with FOLFIRINOX, liver metastasis, IT with pembrolizumab (stable disease after 30 months)	
Overman, 2020 [42]	0/2	NS	NA	NA	NA	NA	NA
Park, 2020 [43]	5/50 ^#^ HRD PDAC	8 as threshold, which can be considered low but was used for correlations with HRD/*BRCA* genes mutations, NS the exact value for each case	NGS with OncoKB / MSK-IMPACT	NA	1 case *KRAS*, *TP53*, *SMAD4*, *BRCA2* biallelic inactivation; 1 case *KRAS*, *TP53*, *CDKN2A*, *BRCA2* biallelic inactivation; 1 case *KRAS*, *SMAD4*, *ARID1A*, *BRCA2* biallelic inactivation: 1 case *KRAS*, *SMAD4*, *BRCA2* biallelic inactivation; 1 case *KRAS*, *TP53*, *CDKN2A*, *SMAD4*.	NA	NA
Salem, 2018 [44]	12/870	17 as threshold, NS the exact value for each case	NGS SureSelect XT assay	5/12 hTMB and MSS, 7/12 MSI	NA	NA	NA
Singhi, 2019 [45]	5/1021	20 as threshold, NS the exact value for each case	Illumina HiSeq technology,	1/5 MSI	One case had both MSI and high-TMB (MLH1 promoter hypermethylation), with *BRAF* p.V600E, other: NS	NA	NA
Tuli, 2019 [46]	1	23.4	NGS (targeted, Foundation Medicine)	MSI	*CHEK2*, *MLH1* mutations	NS	22 months OS
TOTAL	Total of 47; 22/2091 ^#^ (1.1%)	Mean value: 37.6	NGS	19/32 MSI *	Of note: *MMR* genes, *POLE*, *HRD*	8 cases received IT, with different responses (6 partial, 2 complete).	

Abbreviations: PDAC: pancreatic ductal adenocarcinoma; N: number; TMB: tumor mutation burden; mut: number of mutations; Mb: megabase; NGS: next-generation sequencing; MMR: mismatch repair; MSI: microsatellite instability; RCT: radio-chemotherapy; CT: chemotherapy; IHC: immunohistochemistry; WGS: whole-genome sequencing; IT: immunotherapy; AT: adjuvant therapy; NAT: neo-adjuvant therapy; GVAX: clinical trial of vaccine therapy with irradiated allogeneic pancreatic tumor cells transfected with the granulocyte-macrophage colony stimulating factor gene; HRD: homologous recombination deficiency; NA: data not available; NS: data not specified in the study. Notes: ^#^ for calculating high-TMB prevalence, to minimize potential risks of bias, only studies reporting high-TMB prevalence (comparing with low-TMB cases) were reported; * for calculating this percentage, the study of Grant, et al. and of Ngo, et al. have not been taken into account to avoid potential biases, since those study were focused on MSI/dMMR cases only. For more clarity, the column with data regarding the number of cases with high-TMB has been maintained also in this table.

## Supplementary Materials

The following are available online at https://www.mdpi.com/article/10.3390/cancers13133119/s1, Figure S1: PRISMA flow-chart of this systematic review.
