# Supplementary figures and images for: Tumor Mutational Burden as a Potential Biomarker for Immunotherapy in Pancreatic Cancer: Systematic Review and Still-Open Questions
Source: Cancers (Basel). 2021 Jun 22;13(13):3119. doi: 10.3390/cancers13133119 (PMC8269341; doi:10.3390/cancers13133119)

**Supplementary Figure 1.** PRISMA flow-chart of this systematic review

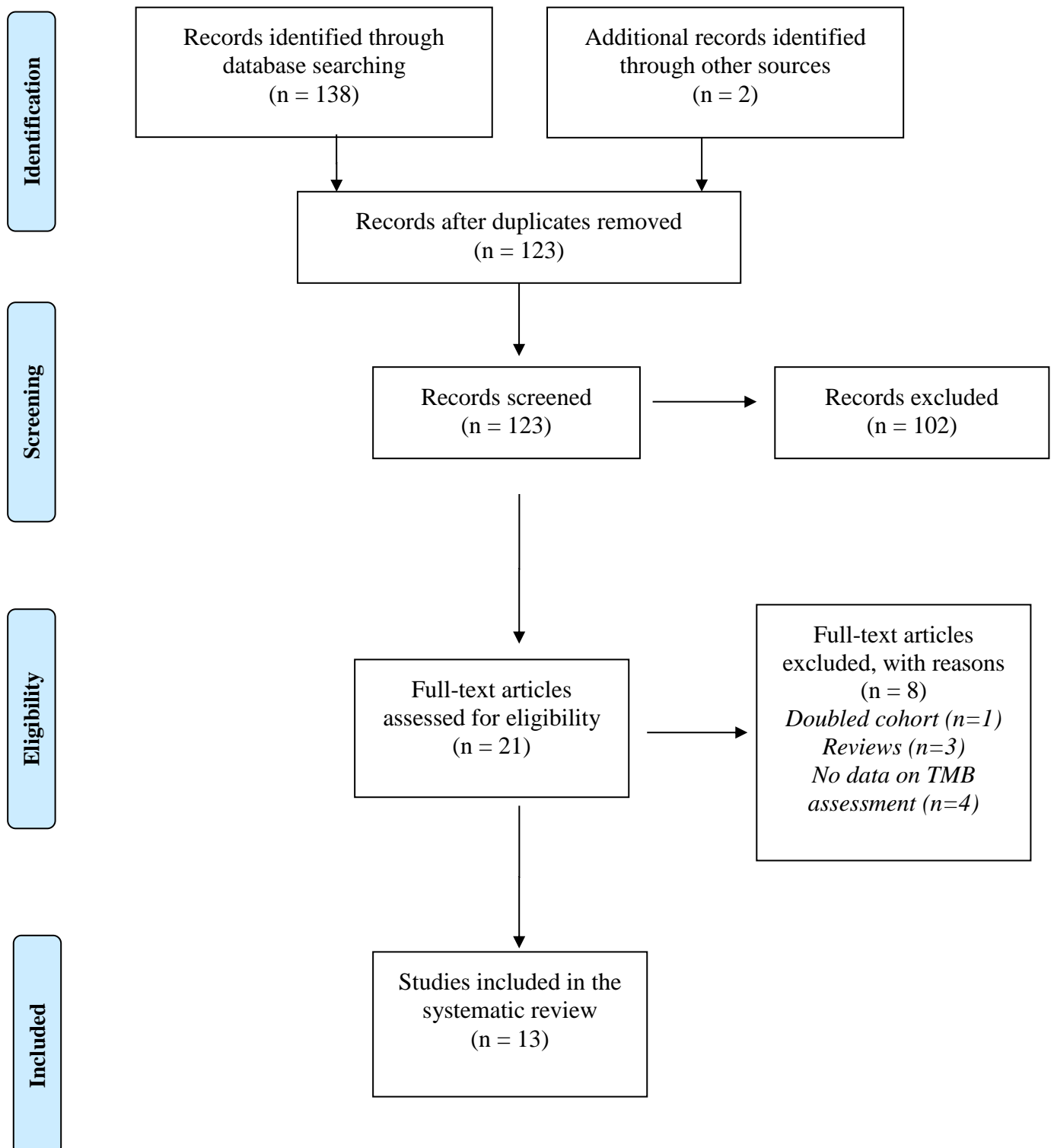

Supplement: Supplementary file 1 [file cancers-13-03119-s001.zip › cancers-1245945-supplementary.pdf]
